# Reversion of Gut Microbiota during the Recovery Phase in Patients with Asymptomatic or Mild COVID-19: Longitudinal Study

**DOI:** 10.3390/microorganisms9061237

**Published:** 2021-06-07

**Authors:** Han-Na Kim, Eun-Jeong Joo, Chil-Woo Lee, Kwang-Sung Ahn, Hyung-Lae Kim, Dong-Il Park, Soo-Kyung Park

**Affiliations:** 1 Medical Research Institute, Kangbuk Samsung Hospital, Sungkyunkwan University School of Medicine, Seoul 03181, Korea; hanna147942@gmail.com (H.-N.K.); chilwoo.lee@gmail.com (C.-W.L.); 2 Department of Clinical Research Design and Evaluation, SAIHST, Sungkyunkwan University, Seoul 03181, Korea; 3 Division of Infectious Diseases, Department of Medicine, Kangbuk Samsung Hospital, Sungkyunkwan University School of Medicine, Seoul 03181, Korea; eunjeong.joo@samsung.com; 4 Functional Genome Institute, PDXen Biosystems Inc., Daejeon 34129, Korea; kwangsung.ahn@gmail.com; 5 Department of Biochemistry, College of Medicine, Ewha Womans University, Seoul 07985, Korea; hyung@ewha.ac.kr; 6 Division of Gastroenterology, Department of Medicine, School of Medicine, Kangbuk Samsung Hospital, Sungkyunkwan University, Seoul 03181, Korea

**Keywords:** SARS-CoV2, COVID-19, asymptomatic, mild, microbiome, gut microbiota, FB ratio

## Abstract

Patients with COVID-19 have been reported to experience gastrointestinal symptoms as well as respiratory symptoms, but the effects of COVID-19 on the gut microbiota are poorly understood. We explored gut microbiome profiles associated with the respiratory infection of SARS-CoV-2 during the recovery phase in patients with asymptomatic or mild COVID-19. A longitudinal analysis was performed using the same patients to determine whether the gut microbiota changed after recovery from COVID-19. We applied 16S rRNA amplicon sequencing to analyze two paired fecal samples from 12 patients with asymptomatic or mild COVID-19. Fecal samples were selected at two time points: during SARS-CoV-2 infection (infected state) and after negative conversion of the viral RNA (recovered state). We also compared the microbiome data with those from 36 healthy controls. Microbial evenness of the recovered state was significantly increased compared with the infected state. SARS-CoV-2 infection induced the depletion of Bacteroidetes, while an abundance was observed with a tendency to rapidly reverse in the recovered state. The Firmicutes/Bacteroidetes ratio in the infected state was markedly higher than that in the recovered state. Gut dysbiosis was observed after infection even in patients with asymptomatic or mild COVID-19, while the composition of the gut microbiota was recovered after negative conversion of SARS-CoV-2 RNA. Modifying intestinal microbes in response to COVID-19 might be a useful therapeutic alternative.

## 1. Introduction

Coronavirus disease (COVID-19) is a respiratory illness caused by a novel coronavirus (severe acute respiratory syndrome coronavirus 2 (SARS-CoV-2)). Since the first case of COVID-19 was reported in Wuhan, China in 2019, more than 60 million people worldwide have contracted the virus as of 1 December 2020. Most patients with COVID-19 have fever, along with respiratory symptoms, and human-to-human transmission usually occurs among close contacts, mainly through respiratory droplets and direct contact. Interestingly, the angiotensin-converting enzyme 2 (ACE2) receptor, used by SARS-CoV-2 to enter the host, is highly expressed in both the respiratory and gastrointestinal (GI) tracts [1,2]; up to 20% of patients have reported GI symptoms such as diarrhea [3,4] and SARS-CoV-2 has been detected in anal swabs and stool samples in almost 50% of patients with COVID-19 [5,6]. Thus, SARS-CoV-2 can damage intestinal epithelial cells and affect commensal gut microbiota.

Studies have shown that respiratory viral infections, such as influenza, are associated with an altered gut microbiome [7,8]. In a recent study that investigated intestinal microbiota alterations in SARS-CoV-2 infection, patients with COVID-19 had significant alterations in fecal microbiomes compared with controls, characterized by the enrichment of opportunistic pathogens and depletion of beneficial commensals [9,10].

However, only hospitalized patients with moderate/severe disease were included in the study and most of them underwent antibiotic or antiviral therapy, which can affect gut microbiota. COVID-19 shows a wide spectrum of severity, ranging from asymptomatic to severe and fatal cases. In our previous study on patients with asymptomatic and mild COVID-19, SARS-CoV-2 showed prolonged shedding in the upper respiratory tract, even in patients with the asymptomatic and mild form of the disease [11]. GI manifestations were observed in 34.7% of patients, and SARS-CoV-2 RNA was detected in the feces of 4.3% of patients [12]. As patients with asymptomatic or mild COVID-19 are common and can also transmit the virus, controlling community transmission is challenging [13]. However, data on SARS-CoV-2 infection and their effect on patients with asymptomatic or mild infection, including gut microbiota profile, are limited. Meanwhile, no detailed longitudinal analysis involving the same patients has been performed to determine if any alterations occur in the composition of gut bacteria after recovery from COVID-19. Here, we aimed to examine the changes in gut microbiota in patients with asymptomatic or mild COVID-19 during recovery and compare their gut microbiome with that of healthy controls.

## 2. Materials and Methods

### 2.1. Study Participants

**Figure 1:**
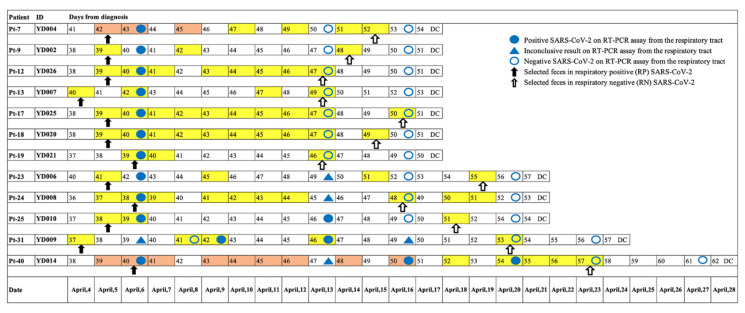
Timeline of the SARS-CoV-2 test results and selection of paired fecal samples of 12 patients. Numbers denote days after diagnosis. Total fecal collection dates are color-coded yellow and positive SARS-CoV-2 results are color-coded orange red. Filled blue circle, filled blue triangle, and empty circle represent a positive SARS-CoV-2 result of respiratory specimen, inconclusive result, and a negative result, respectively. Filled arrow and empty arrow denote paired fecal samples, feces in respiratory positive SARS-CoV-2, and feces in respiratory negative SARS-CoV-2, respectively. DC, discharge.

This prospective study involved 12 patients diagnosed with asymptomatic or mild COVID-19, quarantined between April 4 and April 24, 2020, at Youngdeok Samsung Living and Treatment Center (LTC) in Gyeonsangbuk-do, Korea. LTC is a center for “out-of-hospital quarantine and care,” and patients were defined as being asymptomatic or having mild COVID-19 when they satisfied all of the following conditions: (1) age under 60 years, (2) no underlying chronic diseases, (3) alert mentality, (4) body temperature below 37.5 °C and (5) no radiological evidence of pneumonia, per the Korea Centers for Disease Control and Prevention severity criteria (http://ncov.mohw.go.kr/en/ (accessed on 5 March 2020)). The 12 patients included in this study were selected from 46 patients in the cohort of a previous study, which evaluated the GI symptoms and fecal viral shedding in asymptomatic or mild COVID-19 [12]; in these, the results of the respiratory specimen could be reliably judged as positive or negative on the day of collection of their fecal specimen. Two paired fecal samples were selected from 12 patients as follows: (1) a fecal sample collected on or before the positive detection of SARS-COV-2 RNA from the respiratory tract, defined as respiratory positive (RP) SARS-CoV-2 and (2) another fecal sample collected on or after the negative conversion for SARS-COV-2 RNA from the respiratory tract, defined as respiratory negative (RN) SARS-CoV-2. Figure 1 shows a detailed timeline of the SARS-CoV-2 test results from the respiratory and fecal samples of the study participants. 

We have previously reported the association of behavioral and metabolic traits with the gut microbiota in a population-based cohort where feces were sampled in 2014 [14,15,16]. This was before the COVID-19 pandemic, and the subjects had never been exposed to SARS-CoV-2. Thus, we used the data of this cohort as healthy controls. The 36 subjects with high read counts (>50,000 reads/sample) and without the use of antibiotics were included in the current analysis to meet the minimum read count (57,007 reads) for the samples of patients with COVID-19.

### 2.2. Collection of Respiratory/Fecal Samples and Clinical Data 

Beginning on 4 March 2020, respiratory samples of all quarantined patients were collected every Monday and tested for SARS-CoV-2 nucleic acid using real-time reverse transcriptase polymerase chain reaction (rRT-PCR) assay. Respiratory samples comprised upper respiratory tract (nasopharyngeal and oropharyngeal swabs) and lower respiratory samples (sputum). Patients were re-tested on the following Wednesday or Thursday, according to the cyclic threshold (Ct) values reported on Tuesday. Only patients with two consecutive negative results on rRT-PCR assay at least 24 to 48 h apart were discharged from the LTC.

Fecal samples were collected by patients themselves using the stool collection kits and refrigerators provided in each room of the LTC. Patients were instructed to collect a stool sample once daily and store it in the refrigerator. The stool samples were delivered to the medical teams every Monday and Thursday, when they moved from their rooms to undertake the rRT-PCR test using respiratory samples. Further details on the collection of respiratory and fecal samples and clinical data are available in our previous study [12].

### 2.3. DNA Extraction, PCR, and Sequencing of the 16S rRNA Gene

DNA was extracted from fecal samples that were stored in Norgen Stool Nucleic Acid Collection and Preservation Tubes (Norgen Biotek Corporation, Thorold, ON, Canada) and was extracted within 1 month using the DNeasy PowerSoil Pro Kit (QIAGEN, Germantown, MD, USA), according to the manufacturer’s instructions. The V3 and V4 regions of the 16S rRNA gene were amplified by PCR with the following primers: 341F 5-barcode-CCTACGGGNGGCWGCAG-3 and 785R 5-GACTACHVGGGTATCTAATCC-3′. High-throughput sequencing was performed on an Illumina MiSeq instrument (Illumina, San Diego, CA, USA) according to the manufacturer’s instructions. The DADA2 [17] plugin of the QIIME2 package (version 2020.8, https://qiime2.org (accessed on 7 October 2020)) [18] was used to perform sequence quality control and construct a feature table of amplicon sequence variants (ASVs). For taxonomic structure analysis, taxonomy was assigned to ASVs using a pre-trained naïve Bayes classifier and the q2-feature-classifier plugin with the database Silva 138 release in the QIIME2 package. We filtered out features that appeared in only one sample based on the suspicion for downstream analysis. 

### 2.4. Statistical Analysis

We calculated the alpha diversity, such as observed feature, Faith’s phylogenetic diversity (PD), Shannon’s index, and Pielou’s evenness, in the QIIME2 package. For paired samples at two time points (RP SARS-CoV-2 and RN SARS-CoV-2), the Wilcoxon signed-rank exact test was used to perform a hypothesis test of alpha diversity in the RStudio (Version 1.3.1073).

To measure beta diversity, we used the UniFrac distance [19] to estimate dissimilarity at the two time points by incorporating the phylogenetic distances between ASVs. The unweighted and weighted UniFrac distances were calculated for the presence or absence and abundance of ASVs, respectively. Non-phylogenetic methods were also used with Bray–Curtis dissimilarities [20] and Jaccard dissimilarity [21] for the determination of the presence or absence and abundance of ASVs, respectively. Pairwise permutational multivariate analysis of variance (PERMANOVA) test with 999 random permutations was employed to determine the significance of differences in the gut microbiota community between RP SARS-CoV-2 and RN SARS-CoV-2 in the QIIME2 package [18]. Principal coordinates were constructed and plotted using the qiime2R package in RStudio.

To compare the relative abundance of taxa between two paired samples (RP SARS-CoV-2 and RN SARS-CoV-2), we used multivariate analysis by linear models 2 (MaAsLin2) to identify taxa that were significantly associated with the infected status of patients with COVID-19. We used the RP SARS-CoV-2 as a baseline, the two time points as a fixed effect, and subject IDs as a random effect in the mixed model.

For the functional inferences of the microbial community, we used the Phylogenetic Investigation of Communities by Reconstruction of Unobserved States 2 (PICRUSt2) (v2.2.0-b) [22] with ASVs according to a recent manual (https://github.com/picrust/picrust2/wiki (accessed on 2 November 2020)). PICRUSt2 predictions were based on the following gene families: Enzyme Classification numbers (EC numbers, as of 21 January 2016) and Kyoto Encyclopedia of Genes and Genomes (KEGG) orthologs (KO) (v77.1). We generated metabolic pathway database (Metacyc) and KEGG pathway abundance predictions from EC-based and KO-based gene family predictions, respectively [23]. The predicted functional profiles were tested for paired samples (RP SARS-CoV-2 and RN SARS-CoV-2) using the Wilcoxon signed-rank exact test in RStudio and visualized using Statistical Analysis of Taxonomic and Functional Profiles (STAMP) version 2.1.3 [24].

Statistical analyses were conducted using the QIIME2 package or R version 4.0.2 with RStudio (Version 1.3.1073), using two-sided tests, with a *p*-value (*p*) < 0.05 or false discovery rate (FDR) adjusted *q* -value (*q*) <  0.05 considered statistically significant. Graphs were created using R packages such as ggplot2. 

## 3. Results

### 3.1. Clinical Characteristics of Patients with Asymptomatic or Mild COVID-19

The clinical characteristics of the 12 patients with asymptomatic and mild COVID-19 are described in Table 1. The median age was 26 y and 66.7% were men. The median number of days from diagnosis to quarantine was 7 d (range, 5.0–18.0 d). The median number of days from diagnosis to fecal sample examination was 38.0 (range, 36.0–41.0). The median length of follow-up period was 53 d (range, 50–62). Medical history of digestive diseases was reported in four (33.3%) patients, and two of four patients had two or more digestive diseases (Table 1). The mean BMI was 23 ± 5.4 kg/m^2^ in 10 patients. None of the patients had a history of taking medicine, including probiotics or antibiotics. 

### 3.2. Gut Microbiota in RP SARS-CoV-2 and RN SARS-CoV-2 Significantly Differ on Measures of Alpha or Beta Diversity

We analyzed the gut microbiota in paired fecal samples (*n* = 24) of the infected state (RP SARS-CoV-2, *n* = 12) vs. recovered state (RN SARS-CoV-2, *n* = 12) from 12 patients diagnosed with COVID-19. The median interval between the two-paired samples was 10 d. After quality filtering, the mean number of reads for the 24 samples was 126,269 ± 42,654 sequences, and 1394 features were used in the final analysis. Biodiversity, including alpha and beta diversity, was evaluated at ASV levels from the feature table. The 16S rRNA sequencing data were rarefied to 57,007 reads per sample with plateau trend for all individuals, indicating that the biodiversity was adequately covered with the applied sequencing depth (Supplementary Figure S1).

**Figure 2:**
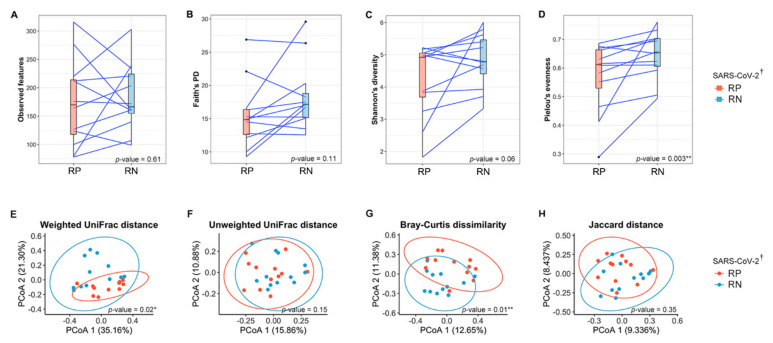
Comparison of the gut microbial diversity between infected (respiratory positive (RP)-SARS-CoV-2) and recovered (respiratory negative (RN)-SARS-CoV-2) states of patients with COVID-19. (**A**) Observed features, (**B**) Faith’s phylogenetic diversity, (**C**) Shannon’s index, and (**D**) Pielou’s evenness were alpha diversity in the paired samples. Blue lines connect pairwise samples of infected and recovered states in same patients. The box plots indicate the interquartile range (IQR). The IQR is the 25th to 75th percentile. The median value is shown as a line within the box. The *p*-values were calculated using Wilcoxon signed-rank exact test. (**E**–**H**) PCoA plots of beta diversity of infected state and recovered state of 12 patients diagnosed with SARS-CoV-2. Overall gut microbial community of patients as represented by PCoA of phylogenetic and non-phylogenetic measurements. (**E**) Weighted UniFrac distance. (**F**) Unweighted UniFrac distance. (**G**) Bray–Curtis dissimilarity. (**H**) Jaccard distance. Each point represents a single sample with ellipses for the 95% confidence interval for each state of COVID-19. The *p*-values were calculated using pairwise permutational multivariate analysis of variance (PERMANOVA) test with 999 random permutations. * *p* < 0.05, ** *p* < 0.01. ^†^ SARS-CoV-2 RNA from the respiratory tract. RP, respiratory positive SARS-CoV-2; RN, respiratory negative SARS-CoV-2.

We defined the RP SARS-CoV-2 as the baseline in all analyses of the present study. Using the number of ASVs observed in each sample (observed features, Figure 2A) and phylogenetic diversity (Faith’s PD, Figure 2B), the richness did not differ significantly between RP and RN (paired Wilcoxon signed-rank exact test; *p* = 0.61 and 0.11 for observed features and Faith’s PD, respectively). However, for evenness, Pielou’s evenness of recovered state (RN SARS-CoV-2) was significantly increased compared with the infected state (paired Wilcoxon signed-rank exact test; *p* = 0.003) (Figure 2D). We also observed that Shannon’s diversity, accounting for both richness and evenness, increased in the infected state in 9 of 12 patients, although this was not statistically significant (paired Wilcoxon signed-rank exact test; *p* = 0.06) (Figure 2C). Supplementary Figure S2 shows the change in alpha diversity based on individuals.

To investigate the dissimilarity of the overall bacterial community between the infection status of COVID-19, we used principal coordinate analysis (PCoA) based on four indices for beta diversity. Considering the abundance of bacteria, we found a significant compositional distinction between the phylogenetic (weighted UniFrac distance, PERMANOVA; *p* = 0.02) and non-phylogenetic (Bray–Curtis dissimilarity, PERMANOVA; *p* = 0.01) indices between the gut microbiota of the infected and recovered state of COVID-19 (Figure 2E,G). The principal components from the weighted UniFrac distance explained 56.46% of the overall variation in the two states of SARS-CoV-2 (Figure 2E). Comparisons of the beta diversity on both phylogenetic (unweighted UniFrac distance) and non-phylogenetic (Jaccard distance) indices using the presence/absence of bacteria between infected and recovered states showed no significant separation by the PCs (PERMANOVA; *p* > 0.05) (Figure 2F,H).

### 3.3. Association among Taxonomy Profiles, Infection Status of COVID-19, and Healthy Controls (HCs)

#### Difference in Taxonomic Composition of Gut Microbiota between RP SARS-CoV-2 and RN SARS-CoV-2 in Patients with COVID-19

**Figure 3:**
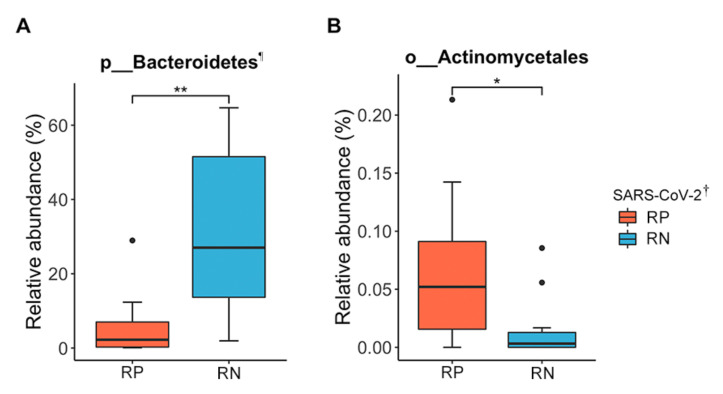
Significantly different taxa profiles of gut microbiota between infected (respiratory positive (RP)-SARS-CoV-2) and recovered (respiratory negative (RN)-SARS-CoV-2) states of patients with COVID-19. (**A**) The relative abundance of phylum Bacteroidetes. ^¶^ The phylum Bacteroidetes, class Bacteroidia, and order Bacteroidales showed the same abundance and statistically significant differences. (**B**) The relative abundance of the order Actinomycetales. The RP-SARS-CoV-2 as a baseline, the two time points as a fixed effect, and subjects’ IDs as a random effect were used in the mixed model using MaAsLin2. * FDR *q* < 0.05, ** FDR *q* < 0.01. ^†^ SARS-CoV-2 RNA from the respiratory tract. RP, respiratory positive SARS-CoV-2; RN, respiratory negative SARS-CoV-2.

Next, we compared the relative abundance of gut microbiota between pairs of SARS-CoV-2 infection status. Firmicutes was the most dominant phylum in the RP SARS-CoV-2 (61.47%), followed by Proteobacteria (25.07%), Actinobacteria (7.45%), and Bacteroidetes (5.80%). Firmicutes was also the most dominant phylum in the RN SARS-CoV-2 (45.78%), followed by Bacteroidetes (31.80%), Proteobacteria (18.61%), and Actinobacteria (3.09%). Among the 13 phyla, 18 classes, 44 orders, 88 families, 234 genera, 1 phylum, 1 class, and 1 order were significant after adjusting for FDR (*q* < 0.05). The phylum Bacteroidetes, along with its sub-taxon class Bacteroidia and order Bacteroidales, were depleted in RP SARS-CoV-2 compared to paired RN SARS-CoV-2, where the relative abundances were 5.8% and 31.8%, respectively. Bacteroidetes increased the odds of the RN SARS-CoV-2 (3.34-fold) compared to the infected state (mixed-effect model; exponentiated coefficient = 3.34, FDR *q* = 9.17 × 10^−4^) (Figure 3A). In contrast, the order Actinomycetales was decreased in RN SARS-CoV-2 compared to RP SARS-CoV-2 (mixed-effect model; exponentiated coefficient = 0.42, FDR *q* = 0.04) (Figure 3B). The relative abundances in the infected and recovered states were 0.07% and 0.02%, respectively. 

Additionally, we found a high abundance of the families Bacteroidaceae, Marinifilaceae, and Tannerellaceae and the genus *Bacteroides* belonging to Bacteroidetes and a low abundance of the family Actinomycetaceae and the genus *Actinomyces* belonging to Actinobacteria in the RN SARS-CoV-2 compared to the RP SARS-CoV-2 when we used the relaxed FDR level (*q* < 0.1) (Supplementary Table S1).

### 3.4. Firmicutes/Bacteroidetes (F/B) Ratio Was Markedly Increased in RP SARS-CoV-2

**Figure 4:**
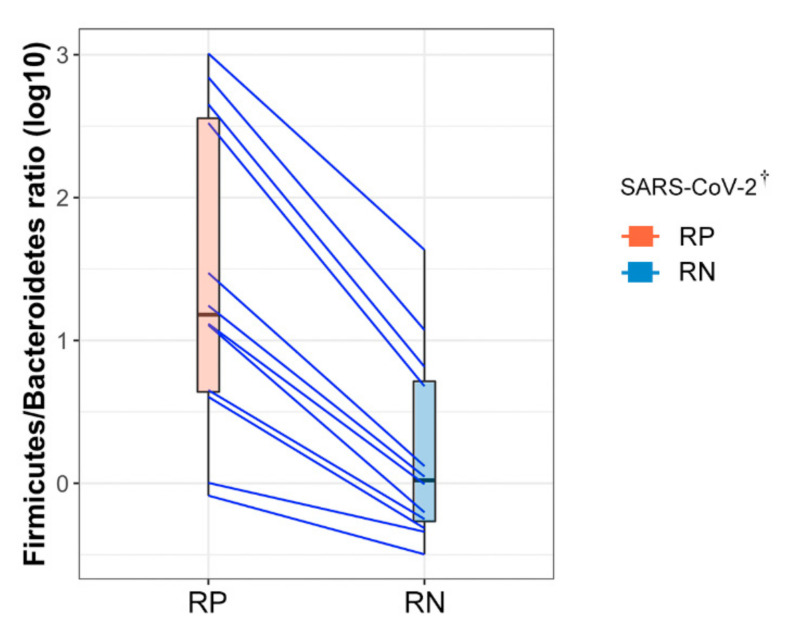
Comparison of Firmicutes to Bacteroidetes ratio in paired samples diagnosed with COVID-19. Each point on the left and right sides of the slope graph corresponds to the samples collected in the infected state (respiratory positive (RP)-SARS-CoV-2) and in the recovered state (respiratory negative (RN)-SARS-CoV-2), respectively. Paired samples from the same subject are connected with a blue line showing the change in the ratio. ^†^ SARS-CoV-2 RNA from the respiratory tract. RP, respiratory positive SARS-CoV-2; RN, respiratory negative SARS-CoV-2.

The F/B ratio was calculated to predict dysbiosis related to COVID-19 infection status. We observed that RP SARS-CoV-2 exhibited a markedly higher F/B ratio than RN SARS-CoV-2 (paired Wilcoxon signed-rank exact test; *p* = 0.042) due to a significant depletion of Bacteroidetes in RP SARS-CoV-2 (Figure 4). The mean F/B ratio was 215.02 ± 341.45 and 6.01 ± 12.19 in feces at the two time points of the 12 patients with COVID-19, respectively. Interestingly, as the F/B ratio imbalance was alleviated by depletion of SARS-CoV-2, the dysbiosis environment appeared to shift to a more symbiotic environment after the negative conversion of SARS-CoV-2, irrespective of the short interval time (median, 10 days: range, 7–17 days) between the collection of RP SARS-CoV-2 and RN SARS-CoV-2.

### 3.5. Comparison of Patients with COVID-19 and HCs

**Figure 5:**
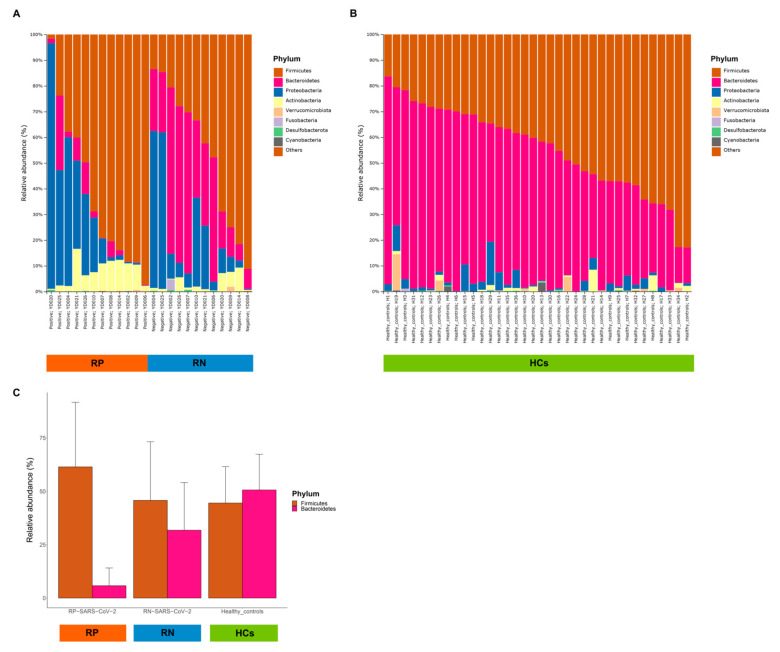
Comparison of taxonomic compositions of gut microbiota at the level of phylum. (**A**) Relative abundance of phyla between two time points (respiratory positive (RP)-SARS-CoV-2 and respiratory negative (RN)-SARS-CoV-2) in 12 patients with COVID-19. (**B**) Relative abundance of phyla in 36 individuals as healthy controls (HCs). (**C**) The mean relative abundance of Firmicutes and Bacteroidetes among patients with COVID-19 and HCs.

**Figure 6:**
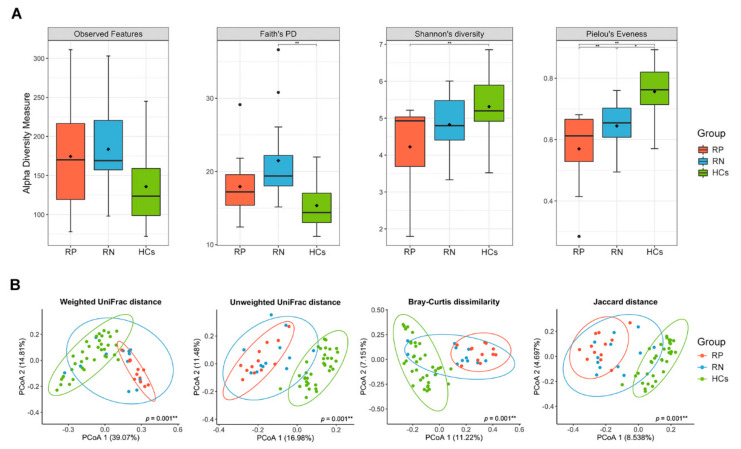
Comparison of the gut microbial diversity among respiratory positive (RP)-SARS-CoV-2, respiratory negative (RN)-SARS-CoV-2, and healthy controls. (**A**) Alpha diversity. The interquartile range (IQR) is the 25th to 75th percentile. The median and mean values are shown as a line and diamond within the box, respectively. The *p*-values were calculated using mixed model. (**B**) Beta diversity. Each point represents a single sample with ellipses for the 95% confidence interval for each group. The *p*-values were calculated using pairwise permutational multivariate analysis of variance (PERMANOVA) test with 999 random permutations. * *p* < 0.05, ** *p* < 0.01. RP, respiratory positive SARS-CoV-2; RN, respiratory negative SARS-CoV-2; HCs, healthy controls.

To investigate whether the gut microbiota of patients with COVID-19 differs from that of healthy individuals, we compared the composition of the gut microbiota among RP SARS-CoV-2, RN SARS-CoV-2 and HCs. Interestingly, a major observation was that the gut microbiota in RN SARS-CoV-2 showed a tendency to return to a healthier composition than in RP SARS-CoV-2 for both taxonomic composition (Figure 5A,B) and biodiversity (Figure 6). We discovered that the evenness of gut microbiota increased with recovery after infection and was at its highest in HCs (Figure 6A). Regarding beta diversity, the gut microbial communities of RP SARS-CoV-2 and HCs were clearly distinguished, and RN SARS-CoV-2 was clustered in the intermediate of the two groups (Figure 6B). In the 36 healthy controls, Bacteroidetes was the most abundant phylum (50.66%), followed by Firmicutes (44.53%), Proteobacteria (2.80%), and Actinobacteria (0.91%), which is similar to the results of 766 subjects reported in a previous study [16]. The relative abundance of Bacteroidetes increased with the recovery from infection of SARS-CoV-2, and this was higher than that of Firmicutes in HCs (Figure 5C). Comparing the RN SARS-CoV-2 and HCs, no difference was observed between the RN SARS-CoV-2 state and HCs with respect to the abundance of Firmicutes, but a significant decrease in Proteobacteria and Actinobacteria abundance and an increase in Bacteroidetes abundance in HCs (Figure 5A,B, and Supplementary Table S2) were observed. 

Additionally, we observed greater changes at lower taxonomic levels between patients with COVID-19 and HCs. The abundance of the genera *Escherichia/Shigella* and *Citrobacter* within Proteobacteria and genera *Collinsella* and *Bifidobacterium* within Actinobacteria was positively correlated with COVID-19 in both RP SARS-CoV-2 and RN SARS-CoV-2 (Supplementary Table S2). The relative abundance of the genera was 1.5- to 11-fold higher than that of HCs. In contrast, the abundance of short-chain fatty acid (SCFA)-producing bacteria, such as members of the genera *Bacteroides*, *Butyricimonas*, and *Odoribacter* within the phylum Bacteroidetes and some genera in the families Lachnospiraceae and Rumminococcaceae, was markedly reduced in patients with COVID-19 compared to the HCs. In the comparison between patients with COVID-19 and healthy controls, most of the taxa showed a greater difference in RP SARS-CoV-2, but the direction of the coefficients was the same in RN SARS-CoV-2, even though some taxa did not differ significantly.

### 3.6. Predicted Metabolic Pathways

**Figure 7:**
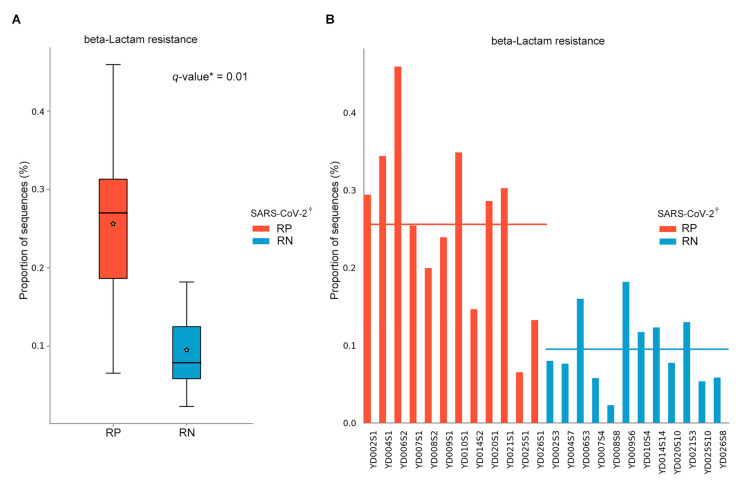
Inferred gut microbiota functions by PICRUSt2 from 16S rRNA gene sequences between infected (respiratory positive (RP)-SARS-CoV-2) and recovered (respiratory negative (RN)-SARS-CoV-2) states of patients with COVID-19. (**A**) The predicted mean relative abundance of beta-lactam resistance (ko00312 from KEGG pathway) between RP-SARS-CoV-2 and RN-SARS-CoV-2. Box plots denote the top quartile, median and bottom quartile, and white stars mean the average value. All the differences were analyzed using Wilcoxon signed-rank exact test followed by Benjamini–Hochberg FDR method, which was used to correct multiple comparisons (* *q* < 0.05). (**B**) The predicted mean relative abundance of beta-lactam resistance in individual-based bar plot in 12 patients with COVID-19. Each red and blue bar is aligned according to the same patient pair. ^†^ SARS-CoV-2 RNA from the respiratory tract. RP, respiratory positive SARS-CoV-2; RN, respiratory negative SARS-CoV-2.

For a better understanding of the function of COVID-19-associated bacteria, we inferred the predictive pathways with the MetaCyc and KEGG database using PICRUSt2. Among the pathways, a KEGG pathway related to beta-lactam resistance (ko00312) exhibited significant differences between the two statuses (mixed-effect model; exponentiated coefficient = 0.64, FDR *q* = 0.01) (Figure 7A). The result was confirmed in individual patients with COVID-19 (Figure 7B); however, no difference existed in results using the MetaCyc database (FDR *q* > 0.05).

## 4. Discussion

In this study, we sought to describe the gut microbiome of patients with COVID-19 and compare the altered gut microbiome between the infected state and negative conversion state of SARS-CoV-2 RNA from the respiratory tract in the same patients. The results demonstrated that the gut microbial diversity and taxonomic composition changed significantly during the recovery phase in asymptomatic or mild COVID-19 patients. Our observations suggest that SARS-CoV-2 infection induces the depletion of Bacteroidetes, whereas the taxon Firmicutes is relatively dominant, leading to dysbiosis in the intestinal environment. Nevertheless, the composition of the gut microbiota in patients with COVID-19 recovered relatively rapidly based on the short interval between positive detection to the negative conversion of the viral RNA in the respiratory tract. We also investigated the gut microbiota in patients with COVID-19 compared with HCs who had never been exposed to SARS-CoV-2. Notably, our results indicated that patients with COVID-19 and HCs could potentially be distinguished by the gut microbiota. Moreover, we found that several SCFA-producing genera were remarkably depleted in patients with COVID-19 compared to HCs.

Greater evenness of gut microbiota was found in RN SARS-CoV-2 than RP SARS-CoV-2 in the same patients with COVID-19. An increase in Bacteroidetes, including their lower taxa, after negative conversion of SARS-CoV-2 RNA might have contributed to the increasing evenness of the gut microbiota.

The gut microbiota of RP SARS-CoV-2 exhibits a markedly higher F/B ratio than that of RN SARS-CoV-2 of patients with COVID-19; this ratio might be a possible biomarker for the negative conversion of viral RNA from the respiratory tract. The human gut microbiota is dominated by two bacterial phyla, Firmicutes and Bacteroidetes, which represent more than 90% of the total community, and this composition remains relatively unaffected by acute perturbations, as its plasticity allows the microbiota to rapidly return to its initial composition [25]. However, gut dysbiosis by various factors has deleterious effects on host health, including the gastrointestinal tract [26] and immune system [27], and metabolic diseases [28]. The F/B ratio is known as a hallmark of obesity, and, interestingly, two recent studies have reported strong evidence for a causal impact of obesity on the susceptibility and severity of COVID-19 [29,30]. However, recent studies have reported no association between the F/B ratio and obesity [31]. The mean BMI was 23.5 kg/m^2^ in our patients, and none had a history of chronic metabolic disease. Thus, the F/B ratio might be independently associated with SARS-CoV-2 infection status in this study.

To date, only very few studies have explored the gut microbiota of COVID-19 patients [9,10,32]. Our results support the previous results of Zuo et al., who found that multiple species from the Bacteroidetes phylum correlated inversely with fecal shedding of SARS-CoV-2 [9]. Although our study included the cohort of asymptomatic and mild disease, the results were comparable to those of Zuo et al., who included only hospitalized patients with moderate/severe disease. They also reported the associations between COVID-19 and species belonging to Actinobacteria. Antibiotic-naïve patients with COVID-19 were enriched in opportunistic pathogens known to cause bacteremia, including *Clostridium hathewayi* and *Actinomyces viscosus*, compared with healthy controls. The *Actinomyces odontolyticus* showed a positive correlation with COVID-19 severity [9]. In another study, the gut microbiome of COVID-19 patients had a significantly higher relative abundance of opportunistic pathogens such as *Actinomyces* and *Streptococcus* as compared to the healthy control group [10]. We also observed the higher relative abundance of the order Actinomycetales, including the family Actinomycetaceae and the genus *Actinomyces*, in RP SARS-CoV-2 compared to the RN SARS-CoV-2. *Actinomyces spp.* is known to cause pulmonary actinomycosis, a bacterial lung infection. In mice, influenza virus infection of lungs caused an increase in the relative abundance of the class Actinobacteria [33]. However, a recent study reported a high abundance of Bacteroidetes and low abundance of Actinobacteria in patients with COVID-19 [32]. Due to the relatively small sample size, these results need to be confirmed in further studies with larger sample size and more techniques.

Recent studies have shown that respiratory viral infections such as Influenza A viruses (IAVs) alter the composition of the gut microbiota [8,34]. IAV infection preferentially depletes Bacteroidetes in the small intestine [8], and these changes account for the enhanced susceptibility to secondary pulmonary bacterial infections [35]. We observed lower proportions of SCFA-producing genera within Bacteroidetes and butyrate-producing genera within Firmicutes in patients with COVID-19 compared with HCs. SCFAs, such as acetate, propionate, and butyrate, are important metabolites in maintaining intestinal homeostasis. Our data are in line with a report demonstrating that depletion of butyrate-producing bacteria reduces the intracellular butyrate/PPARγ signaling, increasing iNOS and nitrate levels, favoring Enterobacteriaceae expansion [36]. Enterobacteriaceae exhibited ≥ 2-fold relative abundance in patients with COVID-19 compared to HCs.

Bacterial metabolites such as SCFAs generated by gut commensals contribute to the maintenance of intact epithelial integrity, regulatory T-cell development, and a relatively anti-inflammatory immune state [7]. SCFAs promote the development of naive CD4+ T cells into regulatory T cells [37]. Clinical studies have shown that although probiotics do not influence the incidence of respiratory tract infection, they reduce the severity of symptoms and duration of the illness [38,39]. A recent report demonstrated that animals fed a high-fiber diet had increased generation of SCFAs, leading to enhanced antiviral CD8+ T cell immune responses and attenuated neutrophil-mediated lung injury during influenza infection, resulting in improved survival [40]. Thus, probiotics including SCFA-producing bacteria can be a treatment modality of COVID-19.

In the PICRUSt2 results of the present study, there were significant differences in the predicted abundance of the β-lactam resistance pathway within asymptomatic and mild patients who had never received any antibiotic treatment. We observed that the proportion of bacterial sequences involved in the β-lactam resistance pathway was increased in RP SARS-CoV-2. Thus far, very limited evidence exists to support patients with COVID-19 contracting secondary bacterial infections. However, despite the relatively low confirmation of secondary bacterial infections, there have been comparatively more reports of antibiotic usage when treating patients with COVID-19 [41], including up to 45% of patients receiving antibiotic treatment [42]. Enterobacteriaceae are β-lactamase-producing bacteria. We identified a large number of Enterobacteriaceae in patients with COVID-19 compared with HCs. For influenza, it has been reported that the infection alters the composition and functionality of the gut microbiota and that these changes account for the enhanced susceptibility to secondary pulmonary bacterial infections [35]. Consistent with influenza, dysbiosis might contribute to secondary bacterial infections in patients with COVID-19. Further studies should identify antimicrobial resistance (AMR) genes correlated with SARS-CoV-2 through shotgun metagenomic sequencing.

The strength of our study is that we observed microbiota changes in patients with asymptomatic or mild symptoms, while SARS-CoV-2 testing in most countries is targeted at patients with symptoms and most enrolled patients in studies were hospitalized. Recovery of dysbiosis might be associated with the severity of COVID-19, as symbiont depletion and gut dysbiosis persisted after the clearance of SARS-CoV-2 in patients with moderate to severe disease [9], while the composition of the gut microbiota was rapidly recovered after negative conversion of SARS-CoV-2 RNA in our patients with asymptomatic and mild COVID-19. Thus, concentrating on disease course and modifying intestinal microbes in response to COVID-19 may represent useful therapeutic alternatives. Additionally, none of the patients received any antibiotic treatment or probiotics during quarantine treatment in LTC. Fecal samples were collected after approximately one month of quarantine, and diet was controlled before and during the study period, as the same meal boxes were provided to every patient. As the gut microbiome is largely influenced by dietary patterns, antibiotics, and probiotics, this cohort, quarantined in LTC, was suitable for gut microbiome analysis since we minimized the influence of other factors. Finally, we performed detailed longitudinal analysis, including diversity and taxonomy, with the same patients to determine any bacterial changes after recovery from COVID-19.

Our study has some limitations. First, the sample size was relatively small, as we only selected patients whose respiratory sample result could be precisely judged as positive or negative on the day of the fecal sample collection. Second, we did not investigate longitudinal changes in the gut microbiota from RP SARS-CoV-2 and RN SARS-CoV-2, except for two time points. However, despite the short interval between RP SARS-CoV-2 and RN SARS-CoV-2, the difference between the two states was evident. Third, we could not investigate the gut microbiota at the diagnostic baseline, as fecal samples were collected at least 38 days following diagnosis. Compositional differences at earlier times might be more useful for disease management and certain species may have already bloomed (or decreased) and disappeared (or increased) during a one-month period. However, our results are meaningful to explain the gut microbiome in prolonged respiratory shedders with asymptomatic or mild COVID-19, who were in quarantine for over a month. Finally, our study was based on 16S rRNA gene sequencing, which provides limited information about bacterial genes and their functions. Whole metagenomic sequencing is in progress, which will expand our understanding of the strains associated with COVID-19, their genes and functions, and metabolic pathways.

The gut microbiota could be a promising target for the management or prevention of COVID-19. Profiling the gut microbiota of patients and recommending an effective diet, including specialized pre/probiotics to improve gut dysbiosis, might improve the overall immune response in patients with COVID-19. SARS-CoV-2 might interfere with host immunity through the depletion of beneficial microbial taxa. It is also worth considering whether the efficacy can be improved by manipulating the gut microbiota during vaccination.

## 5. Conclusions

Gut dysbiosis was observed after infection even in patients with asymptomatic or mild COVID-19, while the composition of the gut microbiota was recovered after negative conversion of SARS-CoV-2 RNA in the respiratory tract. The pathogenic role of dysbiosis should be demonstrated in further studies and altering the gut microbiota might be a promising target for the management and prevention of COVID-19.

## Figures and Tables

**Table 1:** Clinical characteristics of enrolled patients.

Characteristic	All Patients (*n* = 12)
Age, median (range)	26 (18–47)
Male, *n* (%)	8 (66.7%)
Days from diagnosis to quarantine, median (range)	7 (5.0–18.0)
Days from diagnosis to fecal sample examination, median (range)	38.0 (36.0–41.0)
Days from diagnosis to last follow up, median (range)	53.0 (50.0–62.0)
Medical history, *n* (%)	
Digestive ^1^, *n* (%)	4 (33.3%)
Others ^2^, *n* (%)	3 (25.0%)
BMI (kg/m^2^), mean ± SD	23 ± 6.4

^1^ Digestive disease included reflux esophagitis (*n* = 3), irritable bowel disease (*n* = 2), and fatty liver (*n* = 1); two of four patients had two or more digestive diseases. ^2^ Other diseases included gout (*n* = 1), thyroid cancer (*n* = 1), and pneumothorax (*n* = 1).

## Data Availability

The data presented in this study are available in the article and supplementary material. The raw sequencing data are available on reasonable request from the corresponding author.

## Supplementary Materials

The following are available online at https://www.mdpi.com/article/10.3390/microorganisms9061237/s1, Figure S1: Rarefaction curve based on alpha diversity metrics, Figure S2: Individual-based alpha diversity variation of gut microbiota in patients with COVID-19, Table S1: Comparison of taxonomic compositions of gut microbiota from the phylum to genus level between respiratory positive (RP)-SARS-COV-2 and respiratory negative (RN)-SARS-COV-2 states, Table S2: Taxonomic compositions of gut microbiota in the respiratory positive (RP)-SARS-COV-2 and respiratory negative (RN)-SARS-COV-2 compared with those in healthy controls, respectively.
