# Supplementary material for: Reversion of Gut Microbiota during the Recovery Phase in Patients with Asymptomatic or Mild COVID-19: Longitudinal Study
Source: Microorganisms. 2021 Jun 7;9(6):1237. doi: 10.3390/microorganisms9061237 (PMC8228238; doi:10.3390/microorganisms9061237)
Supplement: Supplementary file 1 [file microorganisms-09-01237-s001.zip › microorganisms-1245041-supplementary.pdf]

Supplemental Files

Reversion of gut microbiota during the recovery phase in patients with asymptomatic or mild COVID-19: a longitudinal study

Han-Na Kim, Eun-Jeong Joo, Chil-Woo Lee, Kwangsung Ahn, Hyung-Lae Kim, Dong-Il Park*, and Soo-Kyung Park*

Supplementary Fig. S1. Rarefaction curve based on alpha diversity metrics.

Supplementary Fig. S2. Individual-based alpha diversity variation of gut microbiota in patients with COVID-19.

Supplementary Table S1. Comparison of taxonomic compositions of gut microbiota from the phylum to genus level between respiratory positive (RP)-SARS-COV-2 and respiratory negative (RN)-SARS-COV-2 states

Supplementary Table S2. Taxonomic compositions of gut microbiota in the respiratory positive (RP)-SARS-COV-2 and respiratory negative (RN)-SARS-COV-2 compared with those in healthy controls, respectively.

* Corresponding authors contact details:

Soo-Kyung Park, Sungkyunkwan University School of Medicine, Seoul, Republic of Korea: skparkmd@gmail.com

Dong Il Park, Sungkyunkwan University School of Medicine, Seoul, Republic of Korea: diksmc.park@samsung.com

Supplementary Fig. S1. Rarefaction curve based on alpha diversity metrics.

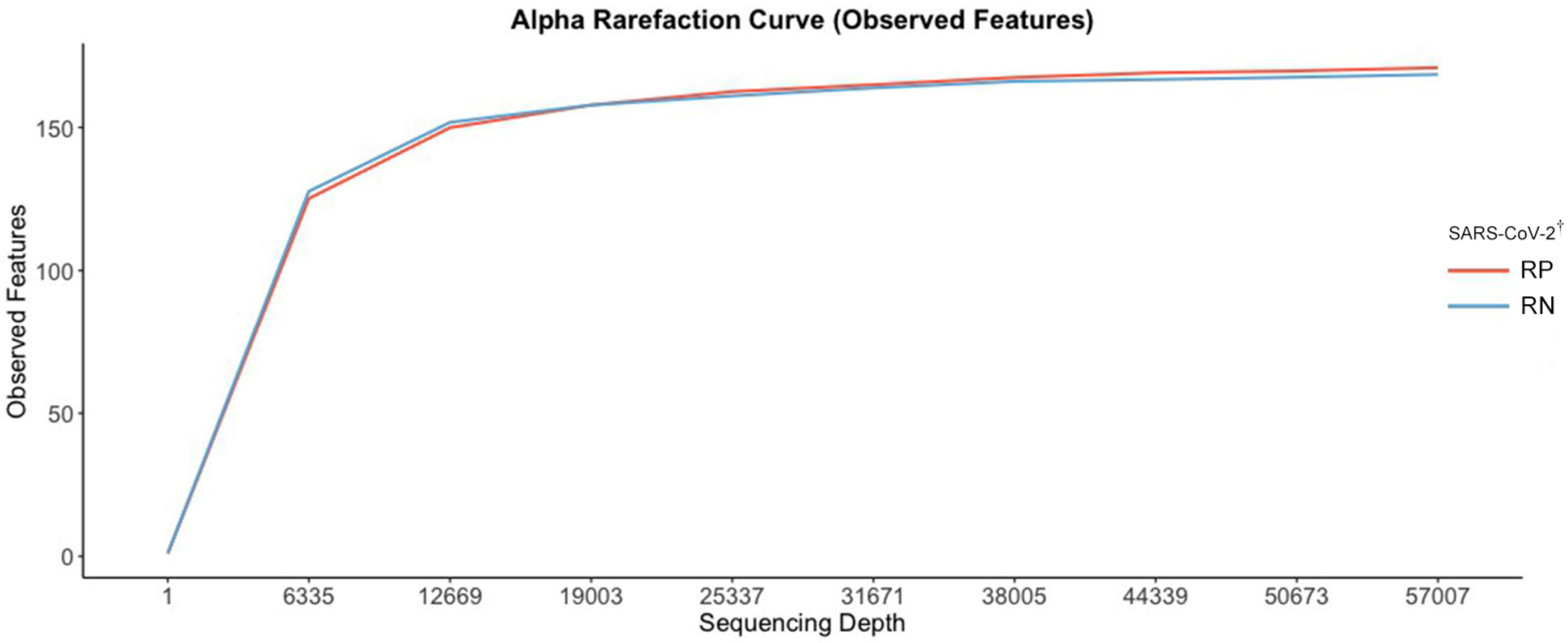

The number of observed ASVs indicated that 57,007 sequences per sample are sufficient for capturing the alpha diversity of microbial communities in both respiratory positive (RP) SARS-CoV-2 and respiratory negative (RN) SARS-CoV-2. The x-axis shows the number of sequences per sample. The rarefaction curves construction (10 replicates/depth) was performed using the "diversity alpha-rarefaction" plugin QIIME2. †SARS-CoV-2 RNA from the respiratory tract. RP, respiratory positive SARS-CoV-2; RN, respiratory negative SARS-CoV-2

Supplementary Fig. S2. Individual-based alpha diversity variation of gut microbiota in patients with COVID-19.

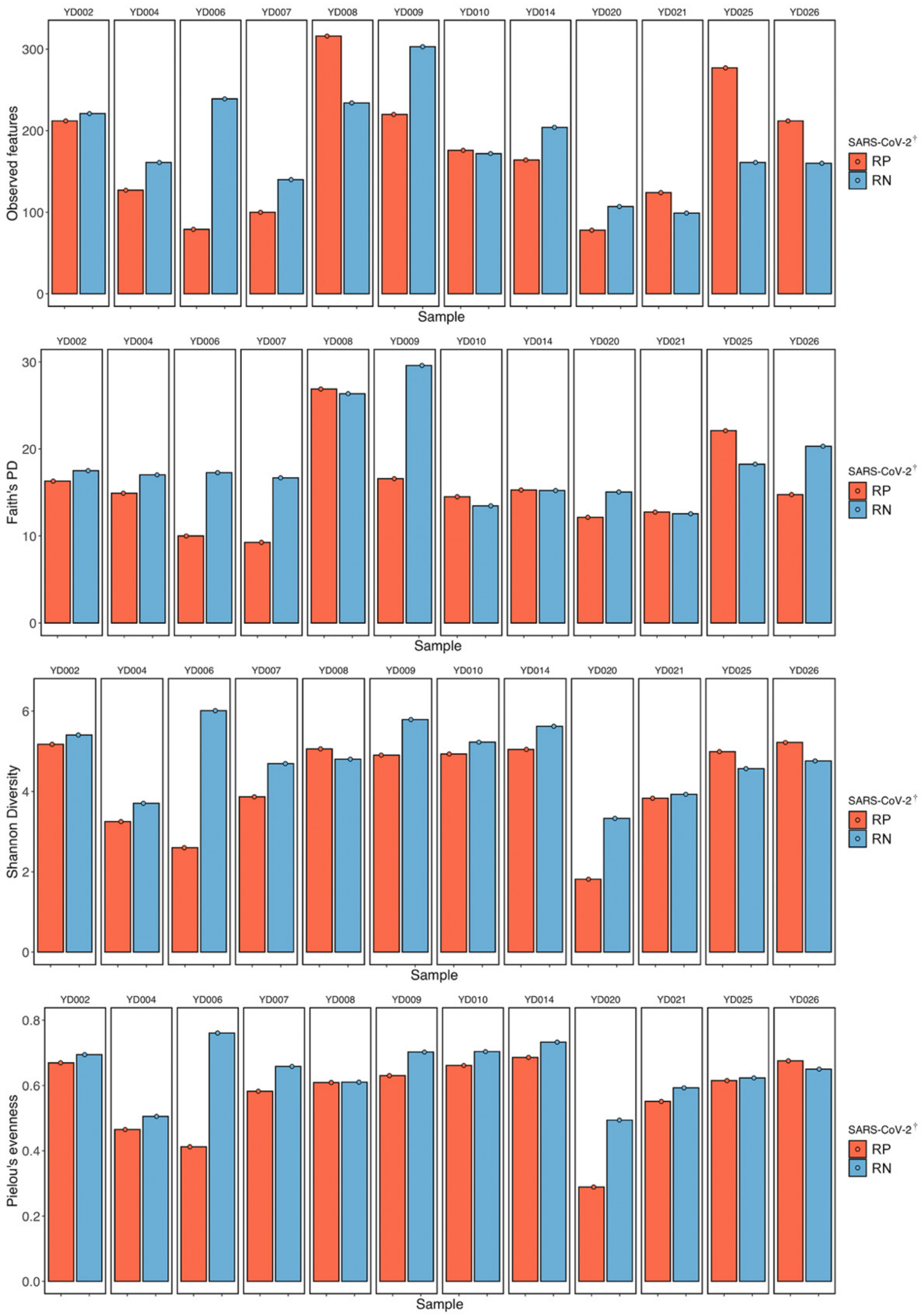

†SARS-CoV-2 RNA from the respiratory tract. RP, respiratory positive SARS-CoV-2; RN, respiratory negative SARS-CoV-2

Supplementary Table S1. Comparison of taxonomic compositions of gut microbiota from the phylum to genus level between respiratory positive (RP)-SARS-CoV-2 and respiratory negative (RN)-SARS-CoV-2 states

Taxonomy level	Feature	Value [a]	Coef. (SE)	Exp(Coef.) [b]	*p*-value	*q*-value [c]
Phylum	**d__Bacteria;p__Bacteroidota**	RN	1.20 (0.26)	3.34	1.3.E-04	**9.2.E-04***
Phylum	d__Bacteria;p__Actinobacteriota	RN	-0.58 (0.22)	0.56	1.5.E-02	5.1.E-02
Order	**d__Bacteria;p__Bacteroidota;c__Bacteroidia**	RN	1.20 (0.26)	3.34	1.3.E-04	**1.3.E-03***
Class	**d__Bacteria;p__Bacteroidota;c__Bacteroidia;o__Bacteroidales**	RN	1.20 (0.26)	3.34	1.3.E-04	**2.9.E-03***
Class	**d__Bacteria;p__Actinobacteriota;c__Actinobacteria;o__Actinomycetales**	RN	-0.86 (0.24)	0.42	4.0.E-03	**4.4.E-02***
Family	d__Bacteria;p__Actinobacteriota;c__Actinobacteria;o__Actinomycetales;f__Actinomycetaceae	RN	-0.86 (0.24)	0.42	4.0.E-03	7.8.E-02
Family	d__Bacteria;p__Bacteroidota;c__Bacteroidia;o__Bacteroidales;f__Bacteroidaceae	RN	1.14 (0.30)	3.12	2.9.E-03	7.8.E-02
Family	d__Bacteria;p__Bacteroidota;c__Bacteroidia;o__Bacteroidales;f__Marinifilaceae	RN	0.94 (0.29)	2.57	8.2.E-03	8.0.E-02
Family	d__Bacteria;p__Bacteroidota;c__Bacteroidia;o__Bacteroidales;f__Tannerellaceae	RN	1.30 (0.43)	3.65	6.4.E-03	8.0.E-02
Genus	d__Bacteria;p__Actinobacteriota;c__Actinobacteria;o__Actinomycetales;f__Actinomycetaceae;g__Actinomyces	RN	-0.85 (0.24)	0.43	4.1.E-03	9.2.E-02
Genus	d__Bacteria;p__Bacteroidota;c__Bacteroidia;o__Bacteroidales;f__Bacteroidaceae;g__Bacteroides	RN	1.14 (0.30)	3.12	2.9.E-03	9.2.E-02
Genus	d__Bacteria;p__Firmicutes;c__Clostridia;o__Lachnospirales;f__Lachnospiraceae;g__Fusicatenibacter	RN	-0.51 (0.14)	0.60	4.2.E-03	9.2.E-02
Genus	d__Bacteria;p__Firmicutes;c__Clostridia;o__Lachnospirales;f__Lachnospiraceae;g__Lachnoclostridium	RN	1.26 (0.36)	3.54	4.6.E-03	9.2.E-02

[a] RP SARS-CoV-2 was used as a baseline
[b] The coefficients (Coef.) were exponentiated because the relative abundance of taxa was log-transformed in the mixed model.
[c] *q*-values less than 0.1 are listed. A q-value less than 0.05 is boldfaced and marked with an asterisk.

Supplementary Table S2. Taxonomic compositions of gut microbiota in the respiratory positive (RP)-SARS-CoV-2 and respiratory negative (RN)-SARS-CoV-2 compared with those in healthy controls, respectively.

Taxonomy level	Feature [a]	Positive [b]			Negative [b]		
		Coef.	Exp (coef.) [c]	*q*-value [d]	Coef.	Exp (coef.) [c]	*q*-value [d]
Phylum	d__Bacteria;p__Bacteroidota	-1.44	0.24	1.24.E-12	-0.32	0.72	6.64.E-02
Class	d__Bacteria;p__Bacteroidota;c__Bacteroidia	-1.44	0.24	9.31.E-13	-0.32	0.72	5.43.E-02
Class	d__Bacteria;p__Firmicutes;c__Negativicutes	-1.06	0.35	3.27.E-04	-0.96	0.38	7.89.E-04
Order	d__Bacteria;p__Bacteroidota;c__Bacteroidia;o__Bacteroidales	-1.44	0.24	2.08.E-12	-0.32	0.72	7.40.E-02
Order	d__Bacteria;p__Firmicutes;c__Clostridia;o__Oscillospirales	-0.76	0.47	5.92.E-06	-0.26	0.77	1.36.E-01
Order	d__Bacteria;p__Firmicutes;c__Negativicutes;o__Veillonellales;Selenomonadales	-1.45	0.23	6.59.E-04	-1.20	0.30	4.22.E-03
Order	d__Bacteria;p__Firmicutes;c__Clostridia;o__Monoglobales	-1.02	0.36	4.22.E-03	-0.78	0.46	3.23.E-02
Family	d__Bacteria;p__Firmicutes;c__Clostridia;o__Oscillospirales;f__Oscillospiraceae	-1.83	0.16	1.65.E-07	-1.14	0.32	3.88.E-04
Family	d__Bacteria;p__Bacteroidota;c__Bacteroidia;o__Bacteroidales;f__Bacteroidaceae	-1.23	0.29	1.80.E-06	-0.14	0.87	5.73.E-01
Family	d__Bacteria;p__Firmicutes;c__Clostridia;o__Oscillospirales;f__Ruminococcaceae	-0.72	0.49	2.62.E-05	-0.16	0.85	3.52.E-01
Family	d__Bacteria;p__Bacteroidota;c__Bacteroidia;o__Bacteroidales;f__Tannerellaceae	-1.39	0.25	6.11.E-05	-0.27	0.76	4.29.E-01
Family	d__Bacteria;p__Bacteroidota;c__Bacteroidia;o__Bacteroidales;f__Marinifilaceae	-1.40	0.25	7.94.E-05	-0.65	0.52	6.52.E-02
Family	d__Bacteria;p__Bacteroidota;c__Bacteroidia;o__Bacteroidales;f__Prevotellaceae	-1.95	0.14	1.71.E-04	-0.97	0.38	6.42.E-02
Family	d__Bacteria;p__Bacteroidota;c__Bacteroidia;o__Bacteroidales;f__Rikenellaceae	-1.34	0.26	3.12.E-04	-0.72	0.49	5.99.E-02
Family	d__Bacteria;p__Firmicutes;c__Clostridia;o__Oscillospirales;f__Butyricicoccaceae	-0.86	0.42	2.83.E-03	-0.48	0.62	1.04.E-01
Family	d__Bacteria;p__Firmicutes;c__Clostridia;o__Monoglobales;f__Monoglobaceae	-1.02	0.36	3.94.E-03	-0.78	0.46	3.10.E-02
Family	d__Bacteria;p__Bacteroidota;c__Bacteroidia;o__Bacteroidales;f__Barnesiellaceae	-1.33	0.26	1.46.E-02	-1.02	0.36	6.17.E-02
Family	d__Bacteria;p__Firmicutes;c__Negativicutes;o__Veillonellales;Selenomonadales;f__Veillonellaceae	-1.17	0.31	1.47.E-02	-0.76	0.47	1.17.E-01

Family	d__Bacteria;p__Firmicutes;c__Clostridia;o__Oscillospirales;f__Eubacterium_coprostanoligenes_group	-0.77	0.46	4.17.E-02	-0.34	0.71	3.65.E-01
Genus	d__Bacteria;p__Bacteroidota;c__Bacteroidia;o__Bacteroidales;f__Bacteroidaceae;g__*Bacteroides*	-1.23	0.29	4.42.E-06	-0.27	0.76	4.35.E-01
Genus	d__Bacteria;p__Firmicutes;c__Clostridia;o__Oscillospirales;f__Oscillospiraceae;g__*UCG 002*	-1.58	0.21	4.42.E-06	-1.39	0.25	4.28.E-05
Genus	d__Bacteria;p__Firmicutes;c__Clostridia;o__Oscillospirales;f__Ruminococcaceae;g__*Faecalibacterium*	-0.82	0.44	7.79.E-05	-0.30	0.74	1.32.E-01
Genus	d__Bacteria;p__Bacteroidota;c__Bacteroidia;o__Bacteroidales;f__Tannerellaceae;g__*Parabacteroides*	-1.39	0.25	1.18.E-04	-0.27	0.76	4.35.E-01
Genus	d__Bacteria;p__Firmicutes;c__Clostridia;o__Oscillospirales;f__Oscillospiraceae;g__*UCG 003*	-1.33	0.26	2.29.E-04	-1.10	0.33	2.12.E-03
Genus	d__Bacteria;p__Firmicutes;c__Clostridia;o__Lachnospirales;f__Lachnospiraceae;g__*Lachnospiraceae_NK4A136_group*	-1.44	0.24	3.83.E-04	-0.86	0.42	2.99.E-02
Genus	d__Bacteria;p__Proteobacteria;c__Gammaproteobacteria;o__Burkholderiales;f__Sutterellaceae;g__*Sutterella*	-1.67	0.19	8.07.E-04	-0.38	0.68	4.35.E-01
Genus	d__Bacteria;p__Firmicutes;c__Clostridia;o__Oscillospirales;f__Oscillospiraceae;g__*NK4A214_group*	-1.30	0.27	1.28.E-03	-1.15	0.32	4.16.E-03
Genus	d__Bacteria;p__Bacteroidota;c__Bacteroidia;o__Bacteroidales;f__Rikenellaceae;g__*Alistipes*	-1.26	0.28	2.00.E-03	-0.63	0.53	1.17.E-01
Genus	d__Bacteria;p__Bacteroidota;c__Bacteroidia;o__Bacteroidales;f__Marinifilaceae;g__*Odoribacter*	-1.03	0.36	2.10.E-03	-0.35	0.71	2.87.E-01
Genus	d__Bacteria;p__Firmicutes;c__Clostridia;o__Lachnospirales;f__Lachnospiraceae;g__*Roseburia*	-0.85	0.43	2.99.E-02	-1.19	0.31	2.75.E-03
Genus	d__Bacteria;p__Firmicutes;c__Clostridia;o__Lachnospirales;f__Lachnospiraceae;g__*Anaerostipes*	-0.75	0.47	8.02.E-03	-0.82	0.44	4.16.E-03
Genus	d__Bacteria;p__Firmicutes;c__Clostridia;o__Oscillospirales;f__Ruminococcaceae;g__*Ruminococcus*	-1.23	0.29	4.43.E-03	-0.71	0.49	9.89.E-02
Genus	d__Bacteria;p__Firmicutes;c__Clostridia;o__Lachnospirales;f__Lachnospiraceae;g__*Lachnoclostridium*	-1.03	0.36	4.49.E-03	0.23	1.26	5.21.E-01
Genus	d__Bacteria;p__Firmicutes;c__Clostridia;o__Lachnospirales;f__Lachnospiraceae;g__*Agathobacter*	-1.00	0.37	5.33.E-03	-0.50	0.60	1.44.E-01
Genus	d__Bacteria;p__Firmicutes;c__Clostridia;o__Lachnospirales;f__Lachnospiraceae;g__*Lachnospira*	-1.20	0.30	5.39.E-03	-0.74	0.48	8.95.E-02
Genus	d__Bacteria;p__Firmicutes;c__Clostridia;o__Oscillospirales;f__Butyricicoccaceae;g__*Butyricicoccus*	-0.81	0.45	5.70.E-03	-0.45	0.64	1.19.E-01
Genus	d__Bacteria;p__Firmicutes;c__Clostridia;o__Monoglobales;f__Monoglobaceae;g__*Monoglobus*	-1.02	0.36	5.92.E-03	-0.78	0.46	3.43.E-02
Genus	d__Bacteria;p__Firmicutes;c__Clostridia;o__Lachnospirales;f__Lachnospiraceae;g__*Fusicatenibacter*	-0.49	0.61	1.19.E-01	-0.86	0.42	7.24.E-03
Genus	d__Bacteria;p__Firmicutes;c__Clostridia;o__Oscillospirales;f__Oscillospiraceae;g__*Colidextribacter*	-1.02	0.36	7.24.E-03	-0.49	0.61	1.80.E-01
Genus	d__Bacteria;p__Bacteroidota;c__Bacteroidia;o__Bacteroidales;f__Prevotellaceae;g__*Paraprevotella*	-1.19	0.31	1.02.E-02	-0.38	0.68	4.09.E-01
Genus	d__Bacteria;p__Firmicutes;c__Clostridia;o__Lachnospirales;f__Lachnospiraceae;g__*Lachnospiraceae_UCG 010*	-1.04	0.35	1.06.E-02	-0.59	0.55	1.39.E-01

Genus	d__Bacteria;p__Bacteroidota;c__Bacteroidia;o__Bacteroidales;f__Marinifilaceae;g__*Butyricimonas*	-1.03	0.36	1.06.E-02	-0.63	0.53	1.19.E-01
Genus	d__Bacteria;p__Firmicutes;c__Clostridia;o__Peptostreptococcales;Tissierellales;f__Anaerovoracaceae;g__*Family_XIII_UCG 001*	-0.83	0.44	1.51.E-02	-0.86	0.42	1.15.E-02
Genus	d__Bacteria;p__Bacteroidota;c__Bacteroidia;o__Bacteroidales;f__Prevotellaceae;g__*Prevotella*	-1.60	0.20	1.38.E-02	-0.59	0.55	3.60.E-01
Genus	d__Bacteria;p__Firmicutes;c__Clostridia;o__Oscillospirales;f__Oscillospiraceae;g__*Oscillibacter*	-0.99	0.37	1.38.E-02	-0.64	0.53	1.19.E-01
Genus	d__Bacteria;p__Firmicutes;c__Clostridia;o__Lachnospirales;f__Lachnospiraceae;g__*Eubacterium_eligens_group*	-0.96	0.38	1.68.E-02	-0.56	0.57	1.53.E-01
Genus	d__Bacteria;p__Firmicutes;c__Clostridia;o__Lachnospirales;f__Lachnospiraceae;g__*Coprococcus*	-0.94	0.39	1.71.E-02	-0.89	0.41	2.48.E-02
Genus	d__Bacteria;p__Firmicutes;c__Clostridia;o__Oscillospirales;f__Oscillospiraceae;g__*UCG 005*	-0.87	0.42	2.30.E-02	-0.73	0.48	5.81.E-02
Genus	d__Bacteria;p__Firmicutes;c__Clostridia;o__Oscillospirales;f__Ruminococcaceae;g__uncultured	-0.77	0.46	2.78.E-02	-0.17	0.84	6.28.E-01
Genus	d__Bacteria;p__Firmicutes;c__Clostridia;o__Christensenellales;f__Christensenellaceae;g__*Christensenellaceae_R7_group*	-1.01	0.36	3.87.E-02	-0.74	0.47	1.25.E-01
Genus	d__Bacteria;p__Bacteroidota;c__Bacteroidia;o__Bacteroidales;f__Barnesiellaceae;g__*Barnesiella*	-1.11	0.33	4.34.E-02	-0.99	0.37	7.55.E-02
Genus	d__Bacteria;p__Firmicutes;c__Clostridia;o__Oscillospirales;f__;Eubacterium;_coprostanoligenes_group;g__*Eubacterium_coprostanoligenes_group*	-0.77	0.46	4.48.E-02	-0.34	0.71	3.67.E-01
Phylum	d__Bacteria;p__Actinobacteriota	1.43	4.19	4.36.E-06	0.87	2.39	3.94.E-03
Phylum	d__Bacteria;p__Proteobacteria	0.76	2.14	7.54.E-03	0.87	2.39	3.39.E-03
Class	d__Bacteria;p__Actinobacteriota;c__Coriobacteriia	1.96	7.12	6.22.E-13	1.54	4.68	6.31.E-10
Class	d__Bacteria;p__Firmicutes;c__Bacilli	1.12	3.06	3.68.E-06	0.78	2.18	7.82.E-04
Class	d__Bacteria;p__Proteobacteria;c__Gammaproteobacteria	0.76	2.14	5.66.E-03	0.87	2.39	1.71.E-03
Order	d__Bacteria;p__Actinobacteriota;c__Coriobacteriia;o__Coriobacteriales	1.96	7.12	1.38.E-12	1.54	4.68	1.40.E-09
Order	d__Bacteria;p__Firmicutes;c__Bacilli;o__Lactobacillales	2.12	8.36	7.20.E-09	1.63	5.13	3.72.E-06
Order	d__Bacteria;p__Firmicutes;c__Clostridia;o__Peptostreptococcales;Tissierellales	0.94	2.56	7.73.E-05	0.32	1.38	1.86.E-01
Order	d__Bacteria;p__Actinobacteriota;c__Actinobacteria;o__Bifidobacteriales	1.26	3.52	1.89.E-03	0.56	1.76	1.71.E-01
Order	d__Bacteria;p__Proteobacteria;c__Gammaproteobacteria;o__Enterobacterales	1.17	3.22	2.98.E-03	0.83	2.28	3.81.E-02
Order	d__Bacteria;p__Firmicutes;c__Clostridia;o__Clostridiales	1.14	3.14	2.01.E-02	0.46	1.58	3.48.E-01
Family	d__Bacteria;p__Actinobacteriota;c__Coriobacteriia;o__Coriobacteriales;f__Coriobacteriaceae	2.40	11.07	1.16.E-13	1.83	6.24	8.59.E-10

Family	d__Bacteria;p__Firmicutes;c__Bacilli;o__Lactobacillales;f__Enterococcaceae	2.27	9.72	6.37.E-08	1.66	5.25	2.62.E-05
Family	d__Bacteria;p__Firmicutes;c__Bacilli;o__Lactobacillales;f__Lactobacillaceae	2.24	9.39	7.22.E-07	1.70	5.47	7.94.E-05
Family	d__Bacteria;p__Actinobacteriota;c__Coriobacteriia;o__Coriobacteriales;f__Eggerthellaceae	1.16	3.20	2.12.E-06	0.86	2.37	2.24.E-04
Family	d__Bacteria;p__Firmicutes;c__Bacilli;o__Lactobacillales;f__Streptococcaceae	1.22	3.39	2.62.E-05	0.73	2.09	9.83.E-03
Family	d__Bacteria;p__Firmicutes;c__Clostridia;o__Peptostreptococcales;Tissierellales;f__Peptostreptococcaceae	1.32	3.73	9.44.E-05	0.64	1.90	6.12.E-02
Family	d__Bacteria;p__Actinobacteriota;c__Actinobacteria;o__Bifidobacteriales;f__Bifidobacteriaceae	1.26	3.52	1.66.E-03	0.56	1.76	1.58.E-01
Family	d__Bacteria;p__Proteobacteria;c__Gammaproteobacteria;o__Enterobacterales;f__Enterobacteriaceae	1.17	3.22	2.55.E-03	0.79	2.20	4.58.E-02
Family	d__Bacteria;p__Firmicutes;c__Clostridia;o__Clostridiales;f__Clostridiaceae	1.14	3.14	1.86.E-02	0.46	1.58	3.52.E-01
Genus	d__Bacteria;p__Actinobacteriota;c__Coriobacteriia;o__Coriobacteriales;f__Coriobacteriaceae;g__*Collinsella*	2.40	11.07	2.86.E-13	1.83	6.24	2.11.E-09
Genus	d__Bacteria;p__Firmicutes;c__Bacilli;o__Lactobacillales;f__Enterococcaceae;g__*Enterococcus*	2.27	9.72	1.57.E-07	1.66	5.25	5.17.E-05
Genus	d__Bacteria;p__Firmicutes;c__Bacilli;o__Lactobacillales;f__Lactobacillaceae;g__*Lactobacillus*	2.19	8.94	2.88.E-06	1.43	4.18	1.25.E-03
Genus	d__Bacteria;p__Firmicutes;c__Bacilli;o__Lactobacillales;f__Streptococcaceae;g__*Streptococcus*	1.25	3.49	4.28.E-05	0.76	2.14	9.01.E-03
Genus	d__Bacteria;p__Firmicutes;c__Bacilli;o__Erysipelotrichales;f__Erysipelotrichaceae;g__*Turicibacter*	1.45	4.26	4.63.E-05	0.64	1.89	6.61.E-02
Genus	d__Bacteria;p__Firmicutes;c__Clostridia;o__Peptostreptococcales;Tissierellales;f__Peptostreptococcaceae;g__*Romboutsia*	1.44	4.22	4.63.E-05	0.70	2.02	3.87.E-02
Genus	d__Bacteria;p__Firmicutes;c__Clostridia;o__Oscillospirales;f__Ruminococcaceae;g__*UBA1819*	0.56	1.74	1.07.E-01	1.44	4.22	4.67.E-05
Genus	d__Bacteria;p__Firmicutes;c__Bacilli;o__Lactobacillales;f__Lactobacillaceae;g__*Pediococcus*	0.90	2.47	8.02.E-03	1.28	3.61	2.29.E-04
Genus	d__Bacteria;p__Firmicutes;c__Clostridia;o__Lachnospirales;f__Lachnospiraceae;g__*Blautia*	0.90	2.45	2.86.E-04	0.24	1.27	3.21.E-01
Genus	d__Bacteria;p__Firmicutes;c__Clostridia;o__Lachnospirales;f__Lachnospiraceae;g__*Eubacterium_hallii_group*	1.28	3.59	8.84.E-04	0.69	1.99	7.18.E-02
Genus	d__Bacteria;p__Proteobacteria;c__Gammaproteobacteria;o__Enterobacterales;f__Enterobacteriaceae;g__*Escherichia/Shigella*	1.41	4.10	9.73.E-04	0.61	1.84	1.39.E-01
Genus	d__Bacteria;p__Actinobacteriota;c__Actinobacteria;o__Bifidobacteriales;f__Bifidobacteriaceae;g__*Bifidobacterium*	1.26	3.52	2.77.E-03	0.56	1.76	1.59.E-01
Genus	d__Bacteria;p__Proteobacteria;c__Gammaproteobacteria;o__Enterobacterales;f__Enterobacteriaceae;g__*Citrobacter*	0.42	1.52	3.21.E-01	1.11	3.03	1.02.E-02
Genus	d__Bacteria;p__Firmicutes;c__Clostridia;o__Clostridiales;f__Clostridiaceae;g__*Clostridium_sensu_stricto_1*	1.14	3.14	1.99.E-02	0.32	1.38	5.16.E-01
Genus	d__Bacteria;p__Firmicutes;c__Clostridia;o__Peptostreptococcales;Tissierellales;f__Peptostreptococcaceae;g__*Intestinibacter*	0.57	1.76	3.39.E-02	0.22	1.24	4.14.E-01

Genus	d__Bacteria;p__Firmicutes;c__Clostridia;o__Peptostreptococcales;Tissierellales;f__Anaerovoracaceae;g__*Family_XIII_AD3011_group*	0.94	2.55	3.43.E-02	0.67	1.96	1.23.E-01

[a] Compared with healthy controls, less abundant taxa and more abundant taxa are shown with blue and red, respectively, in patients with COVID-19.
[b] Healthy controls were used as a baseline
[c] The coefficients were exponentiated because the relative abundance of taxa was log-transformed in the mixed model.
[d] The *q*-values representing less than 0.05 in RP or RN are listed.
